# Evaluating contaminated land and the environmental impact of oil spills in the Niger Delta region: a remote sensing-based approach

**DOI:** 10.1007/s10661-025-14584-4

**Published:** 2025-09-26

**Authors:** Seyi Adewale Adebangbe, Deborah P. Dixon, Brian Barrett

**Affiliations:** School of Geographical & Earth Sciences, University of Glasgow, Glasgow, UK

**Keywords:** Oil pollution, Environmental degradation, Vegetation health indices, Cloud computing, Machine learning, Niger Delta

## Abstract

The Niger Delta region of Nigeria is a major oil-producing area which experiences frequent oil spills that severely impacts the local environment and communities. Effective environmental monitoring and management remain inadequate in this area due to negligence, slow response times following oil spills, and difficulties regarding access and safety. This study investigates the pervasive issue of oil spills in the Niger Delta region, by employing a remote sensing approach, leveraging geospatial cloud computing and machine learning to evaluate vegetation health indices (SR, SR2, NDVI, EVI2, GRNDVI, GNDVI) derived from PlanetScope satellite data. These indices were analysed using Slow Moving Average regression, which revealed significant declines in vegetation health following oil spill events. The contaminated landcovers exhibit a Spearman’s correlation coefficient (ρ) ranging from − 0.68 to − 0.82, *P* < 0.005 and *P*-values below 0.05 in most landcover categories, suggesting a clear and consistent downward trend in the indices’ values, reflecting a decrease in vegetation health in contaminated areas between 2016 and 2023. A random forest classifier further quantified the extent of contaminated land cover, demonstrating the effectiveness of this method for monitoring environmental damage in this challenging terrain. Contaminated vegetation, wetland, farmland, and grassland cover approximately 4% (1180 ha) of the total Niger Delta area. This integrated approach will enable decision-makers, including government agencies and oil companies, to gain a deeper understanding of the environmental consequences of oil pollution and implement targeted mitigation and remediation strategies.

## Introduction

Nigeria’s Niger Delta, a critical hub for the nation’s oil industry, faces significant environmental challenges due to decades of crude oil exploration (Olukaejire et al., 2024; Steyn, 2009). Frequent oil spills, stemming from aging infrastructure, inadequate maintenance, and oil theft, have released vast quantities of hydrocarbons and heavy metals into the delicate ecosystem, severely impacting both the environment and human health (Muhammad et al., 2024; Olukaejire et al., 2024; Wekpe et al., 2024). Persistent contamination from oil spills has resulted in a cascade of environmental issues, including soil degradation, vegetation deterioration, contamination of surface and groundwater resources, and air pollution (Ekhator et al., 2023; Obi, 2023; Ola et al., 2024).

Remote sensing technologies have emerged as powerful tools for monitoring and assessing environmental impacts, particularly in the context of oil spills (Fingas & Brown, 2014; Löw et al., 2021). Various sensors, including visible, infrared, and radar systems, have been used to detect and quantify the spatial extent of oil spills and their effects on vegetation (Asif et al., 2022; Obida et al., 2021). Biophysical and biochemical parameters affect the spectral reflectance of plants. Healthy green vegetation has a unique profile due to its reflectance peak in the visible range (400–700 nm), a plateau in the near-infrared range (700–1300 nm), and two substantial peaks in the short-wave-infrared range (1300–2500 nm) (Lassalle et al., 2020). The hydrocarbons influence the root structure and capacities of plants, causing them to undergo biophysical and metabolic alterations (Gholizadeh & Kopačková, 2019). Several studies have employed remote sensing and vegetation indices (VIs) to evaluate the impacts of oil spills on vegetation and monitor their recovery (e.g. Adamu et al., 2015, 2018; Obida et al., 2018, 2021; Tucker, 1979; Wakil et al., 2021). Adamu et al. (2018) calculated five VIs from Landsat data, including normalised difference vegetation index (NDVI), soil adjusted vegetation index (SAVI), adjusted resistant vegetation index 2 (ARVI2), green/near-infrared ratio (G/NIR), and green/shortwave infrared ratio (G/SWIR) to differentiate between vegetation conditions at spill sites and non-spill sites. Balogun et al. (2020) used Landsat 8-OLI imagery and machine learning to analyse oil spill impacts on coastal vegetation and wetlands, demonstrating the sensitivity of multiple VIs, such as NDVI, chlorophyll vegetation index (CVI), modified difference water index (MDWI), and green chlorophyll vegetation index (GCVI) in assessing vegetation and wetland stress. Ansah et al. (2022) investigated the effects of oil spills on land cover and identified oil spill hotspots using VIs. They found that enhanced vegetation index (EVI), NDVI, and SAVI were particularly sensitive to oil spill effects on vegetation cover. Similarly, Adamu et al. (2015) examined the variations in 12 broadband multispectral vegetation indices (BMVIs) between pre- and post-spill observations, showing substantial differences. A decline in NDVI values over time in the Niger Delta region due to oil spill impacts was observed by Wakil et al. (2021). They demonstrated a dynamic decrease in NDVI from the 1970 s to 2010, with a slight recovery in 2020. Additionally, Adamu et al., (2016a, 2016b) found that the volume and timing of oil spills can impact VIs extracted from polluted environments. They observed that higher oil spill volumes and shorter time intervals between the spill and image acquisition influence VIs, indicating greater vegetation impact and enhanced detection. Overall, the use of VIs, such as NDVI, SAVI, ARVI2, G/NIR, and G/SWIR, have been found effective in assessing the impacts of oil spills on vegetation and monitoring their recovery. These VIs provide valuable insights into vegetation stress caused by oil spills and can track the recovery trend of contaminated vegetation and wetlands.

Machine learning algorithms have further enhanced the capabilities of remote sensing analysis, enabling more accurate land cover classification and change detection (e.g. Alshari et al., 2023; Loukika et al., 2021; Talukdar et al., 2020; Yuh et al., 2023). Geospatial cloud platforms, such as Google Earth Engine (GEE), have significantly advanced environmental research by offering tools for analysing and visualising large datasets. GEE provides a cloud-based platform for planetary-scale geospatial analysis and has been instrumental in addressing societal challenges such as deforestation, drought, and climate change (Gorelick et al., 2017). The platform’s ability to handle petabyte scale datasets and conduct large-scale computations quickly has made it an essential tool for researchers without access to high-performance computing resources. In recent years, studies have demonstrated the utility of GEE in diverse fields, including environmental monitoring and disaster prediction. For instance, Li et al. (2022) utilised GEE and long short-term memory (LSTM) modeling to forecast dengue outbreaks, highlighting the platform’s ability to handle spatial data for epidemic prediction. Similarly, Ghorbanian et al. (2020) employed GEE to produce a high-resolution land cover map using Sentinel-1 and Sentinel-2 data, demonstrating the platform’s efficacy in handling big satellite data.

Several studies have utilised remote sensing technologies, including optical and radar imagery, to evaluate the impacts of oil spills on vegetation in the Niger Delta. For instance, Kuta et al. (2025) employed Landsat imagery, analysing the NDVI to understand how vegetation response varied by spill volume, timing, and vegetation type. They found significant differences in NDVI responses across vegetation types, with sparse vegetation showing the strongest response, though these impacts diminished over time. Similarly, O’Farrell et al. (2025) specifically targeted mangrove ecosystems using Sentinel-1 Synthetic Aperture Radar (SAR) and machine learning methods, successfully identifying mangrove mortality and unreported spill sites despite inherent challenges in SAR interpretation.

Further work by Ozigis et al., (2019) tested multi-frequency SAR (L-, C-, X-bands), optical sensors (Sentinel-2, Landsat 8), and data fusion, employing machine learning techniques. Their findings highlighted the effectiveness of SAR data, particularly during the wet season, but also acknowledged difficulties in differentiating oil pollution from waterlogging, noting that data fusion benefits were context specific. Likewise, studies by Egobueze et al. (2022) and Adamu et al., (2016a, 2016b, 2018) utilised multiple vegetation indices derived from optical sensors (NDVI, SAVI, ARVI2, G-NIR, G-SWIR, and NDWI) to differentiate spill from non-spill areas and explored temporal detection limitations, consistently identifying constraints linked to cloud cover and declining detectability over time. Balogun (2020) further underscored the limitations of radar and optical data, particularly in accurately distinguishing oil pollution from wet soil and coping with cloud-induced constraints.

Collectively, these studies underscore the utility of remote sensing in monitoring and quantifying oil spill impacts, while highlighting persistent methodological challenges, particularly regarding sensor-specific limitations, cloud interference, difficulties differentiating spill-induced vegetation stress from other environmental stressors, and insufficient integration of multiple data types to comprehensively assess all affected landcover categories.

This study addresses these gaps by proposing an integrated methodological approach that combines cloud free high spatial-resolution PlanetScope imagery, machine learning, and cloud computing. Unlike previous research focusing on limited vegetation types or restricted spatial scales, and minimal coverage due to cloud cover challenges, this study aims to evaluate vegetation health impacts across all major land cover types (including, dense vegetation, grasslands, farmlands, and wetlands) in Bayelsa, Delta, and Rivers states. Specifically, this integrated approach aims to:

- analyse spatio-temporal trends in vegetation health across extensive oil spill-affected regions and
- map the detailed spatial extent and distribution of oil spill contamination across diverse landcover classes.

The integration of high-resolution optical data, advanced analytics, and cloud processing within this approach is designed to provide a robust approach for quantifying oil spill impacts on vegetation. The insights gained into spatial extent and vegetation health trends will ultimately enhance the environmental assessment capabilities available to decision-makers, facilitating the implementation of more targeted and effective mitigation and remediation strategies for oil spill-induced vegetation degradation in the Niger Delta.

## Materials and methods

### Study area

**Fig. 1:**
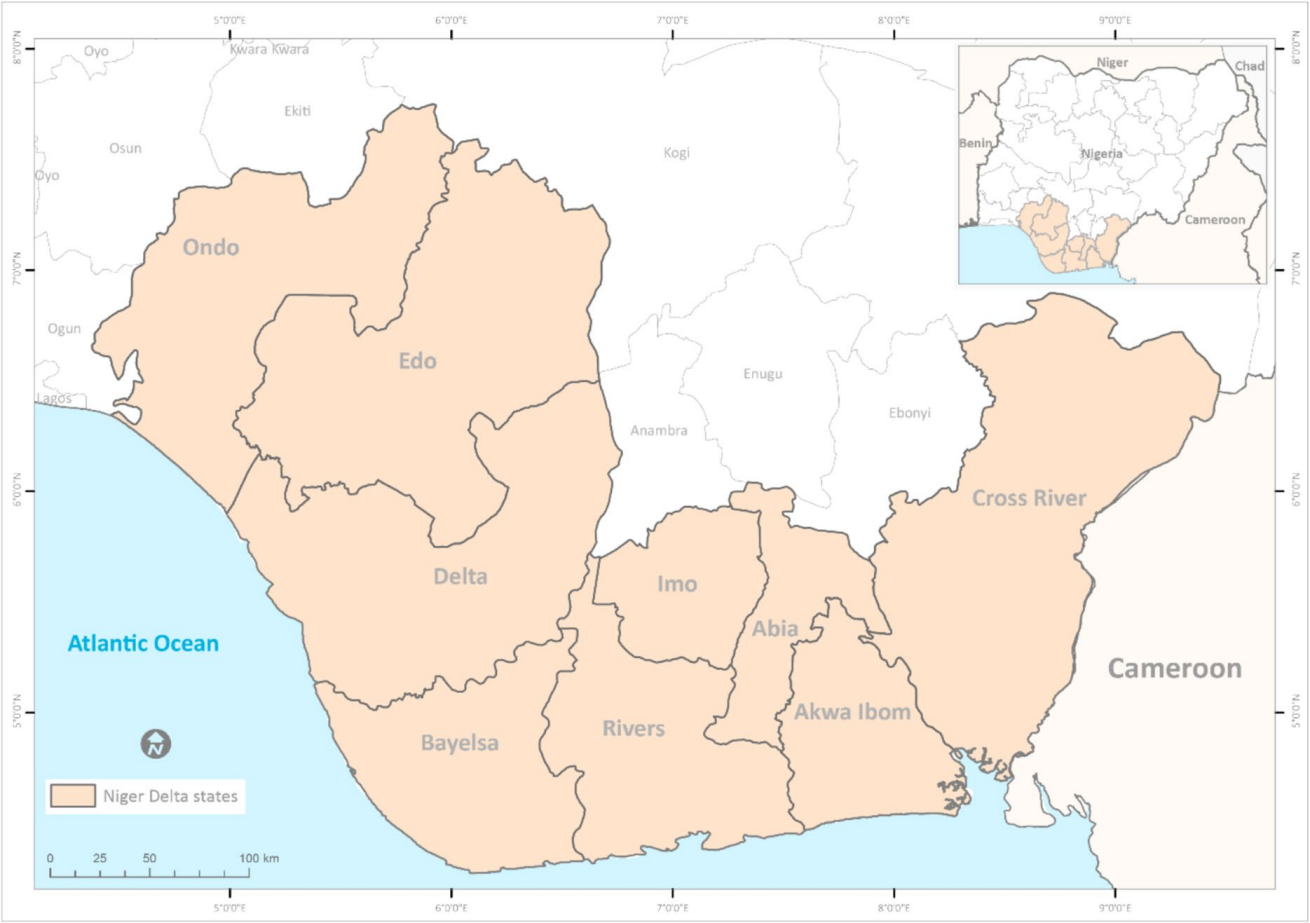
Location of the Niger Delta study area, Nigeria

The Niger Delta, located in Nigeria (see Fig. 1), is one of the world’s largest deltas. It is the largest in Africa and the third largest globally, covering an area of 112,106 km^2^, which accounts for approximately 7.5% of Nigeria’s total landmass (Clinton & Chinago, 2019; Oyegun et al., 2023; Ukhurebor et al., 2021). The delta is a vast, low-lying sedimentary environment, with topography that gently slopes southward towards the Atlantic Ocean. Formed by the Niger River and its tributaries, part of a drainage system exceeding 5000 km in length and encompassing a 2,117,700 km^2^ catchment area, the delta extends approximately 240 km south from Onitsha to the Atlantic coast at the tip of the Nun River. The region is a biodiversity hotspot, hosting Africa’s largest freshwater swamp and a rich variety of plant and animal species. It is also the largest wetland in Africa (Oyegun et al., 2023).

The region comprises nine states: Cross River, Edo, Delta, Abia, Imo, Bayelsa, Rivers, Akwa Ibom, and Ondo. Although oil is extracted in all nine states, this study focuses on the three most affected by pipeline vandalism and oil spills: Bayelsa, Delta, and Rivers. These states account for over 85% of oil spills recorded by the National Oil Spill Detection and Response Agency (NOSDRA).

### Data

Three datasets were used in this study: oil spill incident data, NICFI Satellite Data Program, and landcover data.

#### Oil spill incident data

This study used oil spill data provided by Nigeria’s National Oil Spill Detection and Response Agency (NOSDRA), the official government agency in charge of keeping such records. The data was acquired from 1994 to 2021 through a series of Joint Investigation Visits (JIV). Representatives from regulatory agencies, the oil firm, the affected community, and the security forces make up the joint investigative team. These include the Nigerian Upstream Petroleum Regulatory Commission, the National Oil Spill Detection and Response Agency (NOSDRA), and the State Ministry of Environment. The joint investigation team investigates the cause(s) of the oil spill and is required to agree on and sign a report that verifies the cause (s). The extensive dataset containing over 16,000 records of reported oil spills was initially acquired. After cleaning and filtering, this dataset was streamlined to approximately 5530 usable records. Each record provided key information such as the date, time, GPS coordinates, estimated length, type, volume, and source of the spill. To utilise this data for vegetation impact analysis, the oil spill locations were spatially overlaid onto the landcover map. This process allowed for the identification of areas within different land cover types that experienced oil spill impacts throughout the 2016–2021 study period. Areas subjected to repeated impacts across these years were specifically prioritised for selecting training sites for image classification and for conducting vegetation degradation trend assessment.

##### NICFI Satellite Data Program

The NICFI Satellite Data Program, supported by Norway’s International Climate and Forests Initiative (NICFI) in collaboration with Planet, offers researchers and scientists free access to detailed, high-resolution PlanetScope satellite basemaps covering tropical areas. The dataset includes historical images from December 2015 through August 2020, provided twice yearly, as well as monthly imagery starting from September 2020. These mosaics are specifically created to support scientific analyses by providing accurate spatial representations while minimising distortions from atmospheric conditions and sensor differences.

The satellite images have a spatial resolution (Ground Sample Distance) of 4.77 m, capturing detailed information across four spectral bands—blue, green, red, and near infrared. They are processed into surface reflectance (SR) data, carefully normalised against Landsat-derived SR standards, to ensure consistency and comparability across different time periods. The SR values are scaled by 10,000 and stored as 16-bit integers, making them particularly suitable for precise vegetation index analyses (Planet.com, 2021). Access to these basemaps is streamlined through GEE using Planet’s API, following a simple registration with the NICFI program.

To ensure that our analysis accurately captures vegetation changes linked to specific factors like oil spills—rather than natural seasonal variations—we adopted a careful temporal sampling strategy. For the period between 2015 and 2019, both available bi-annual images were analysed to maintain a balanced seasonal perspective. From 2020 onward, we focused specifically on imagery from July (peak vegetation growth) and December (lowest vegetation period), which helped isolate meaningful vegetation trends from typical seasonal fluctuations.

#### Landcover data

**Fig. 2:**
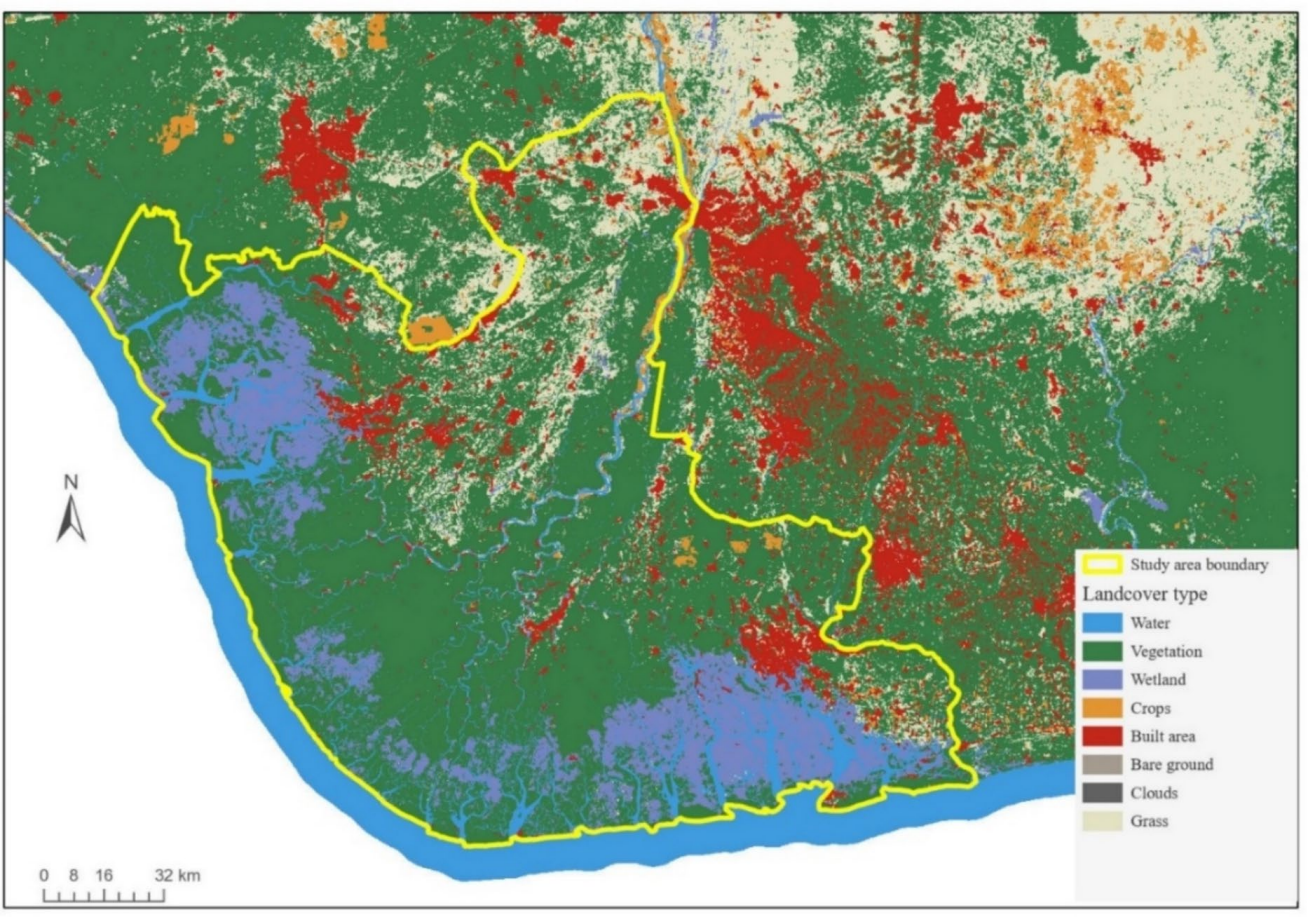
Landcover map of the study area (Delta, Bayelsa, and Rivers states). Data source: Sentinel-2 10 m land use/land cover map (Karra & Kontgis, 2021)

The Sentinel-2 10 m land use/land cover map (Karra & Kontgis, 2021) was used for the landcover classification of the study area (see Fig. 2). The 10-m resolution land use/landcover map was created through a collaboration between Esri, Impact Observatory, and Microsoft. The map was produced using a deep learning model trained on over five billion hand-labelled Sentinel-2 pixels from 20,000 sites across the world’s major biomes. The model uses six bands of Sentinel-2 L2A surface reflectance data (visible blue, green, red, near infrared, and two shortwave infrared bands) and contains nine classes for different landcover categories, including built-up areas, waterbody, and vegetation types. From the landcover map, water, vegetation, wetland, crops/farmland, built-up area, grassland, and cloud were identified.

### Methodology

**Fig. 3:**
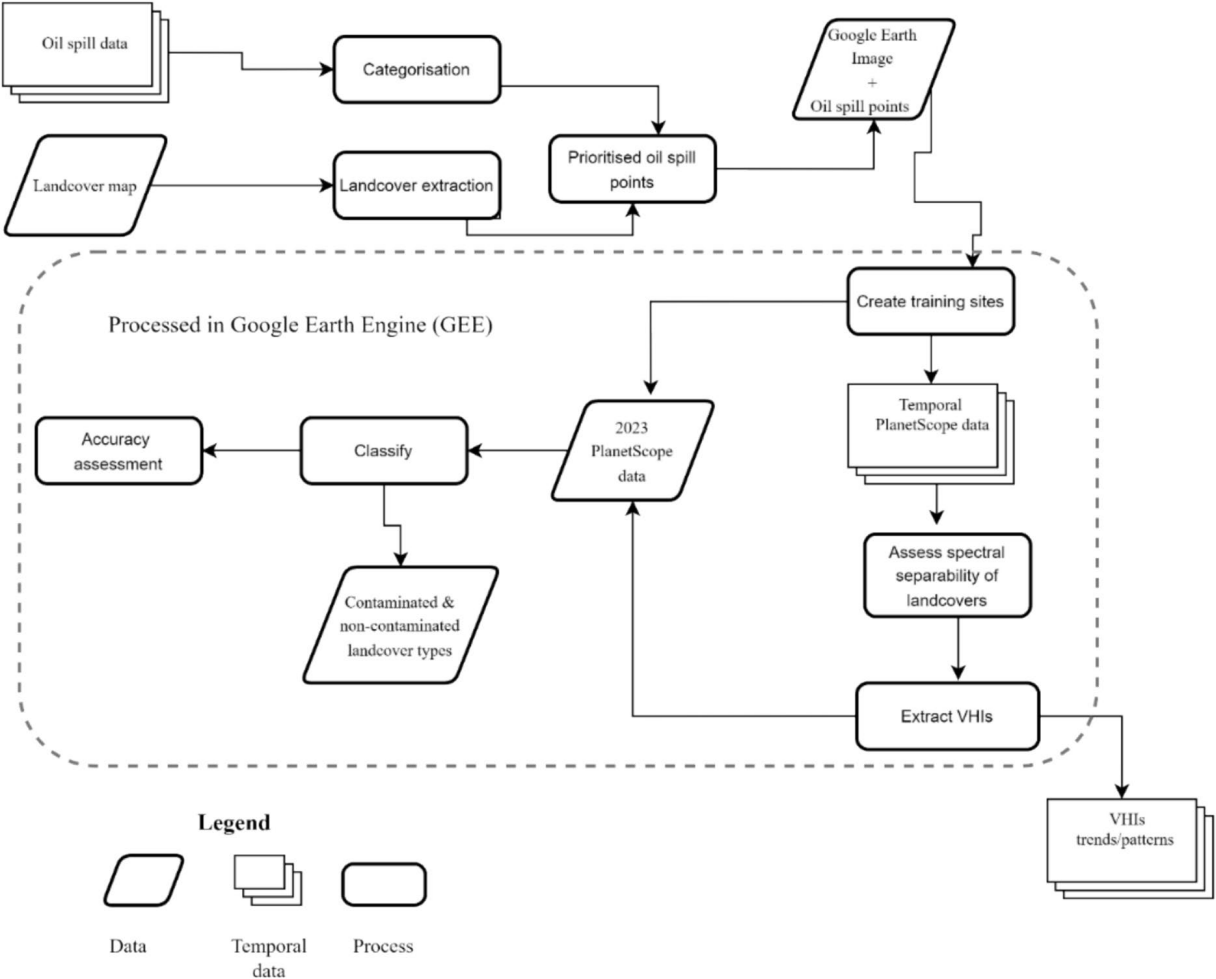
Methodology flowchart

The study’s methodology followed a structured, sequential approach, as illustrated in Fig. 3. First, oil spill data was categorised and prioritised according to the landcover types affected and extent of spills. Subsequently, the corresponding landcover areas were extracted, along with the associated oil spill points. These points were then overlaid onto high-resolution Google Earth imagery, facilitating the generation of training site shapefiles. These shapefiles were subsequently ingested into GEE. Within GEE, a series of pre-processing steps were implemented to prepare the input data for the classifier algorithms. These pre-processing steps encompassed an assessment of the separability of the various landcover types, as well as the extraction of vegetation health indices (VHIs). Following pre-processing, the different input datasets were classified, and the accuracy of the classification was assessed. Both the VHI extraction and the classification sections of the workflow were executed within the GEE environment.

#### Oil spill points sampling

The study examined information on oil spills, including their volume, size, and date, to track spill intensity across different types of landcover. To categorise the oil spill data, a histogram was used with a 100 bbl (barrel) bandwidth based on estimated spill volume. Training site selection prioritised areas significantly impacted by oil spills. Points with estimated spill quantities exceeding 1000 bbl were included, consistent with the methodology of Ozigis et al. (2019). This approach ensured that the spectral characteristics of the selected pixels were representative of typical oil pollution effects on vegetation, providing a strong basis for model training, testing, validation, and subsequent image classification. Complementing this, Adamu et al., (2016a, 2016b) found that spill volumes between 400 and 1000 bbl yielded the most detectable influence on vegetation, evidenced by strong negative relationships with visible spectrum reflectance (Vis).

Assigning oil spill incidents to the appropriate land cover categories is a crucial aspect of the method. The classification process, which utilises a random forest classifier, depends heavily on the spectral signatures obtained from training sites to develop a reliable model. Specifically, spill incidents situated within each land cover class were identified, and these locations served as the training and validation sites for identifying polluted land cover classes impacted by oil spills. The image classification process involved three steps: establishing training datasets, classification, and accuracy assessment. The eight land use and land cover (LULC) classes in the study area were identified, including farmlands/croplands, dense vegetation, grassland, bare lands, built-up areas, water bodies, wetlands, and contaminated areas. To create the training datasets, the LULC classes were converted to polygons using ArcGIS Pro 3.3 and then exported to Google Earth. The contaminated areas were identified by overlaying prioritised oil spill points on the landcover polygons, and vectorised polygons were generated from the affected part of the landcover classes to create the training sites. As demonstrated in previous studies (e.g. Balogun et al., 2020; Obida et al., 2018; Ozigis et al., 2019), non-polluted training sites were also selected for each landcover class, and proximity analysis was employed to ensure they were at least 2000 m away from polluted sites. Google Earth was also used to identify healthy vegetation for the non-contaminated training sites. At least 100 training sites were generated for each of the landcover types, including both contaminated and non-contaminated areas. These training datasets were then loaded into Google Earth Engine for image classification. A median reducer (ee.Reducer.median) was applied to get the median value for each of the over 100 polygons in the different classes.

**Fig. 4:**
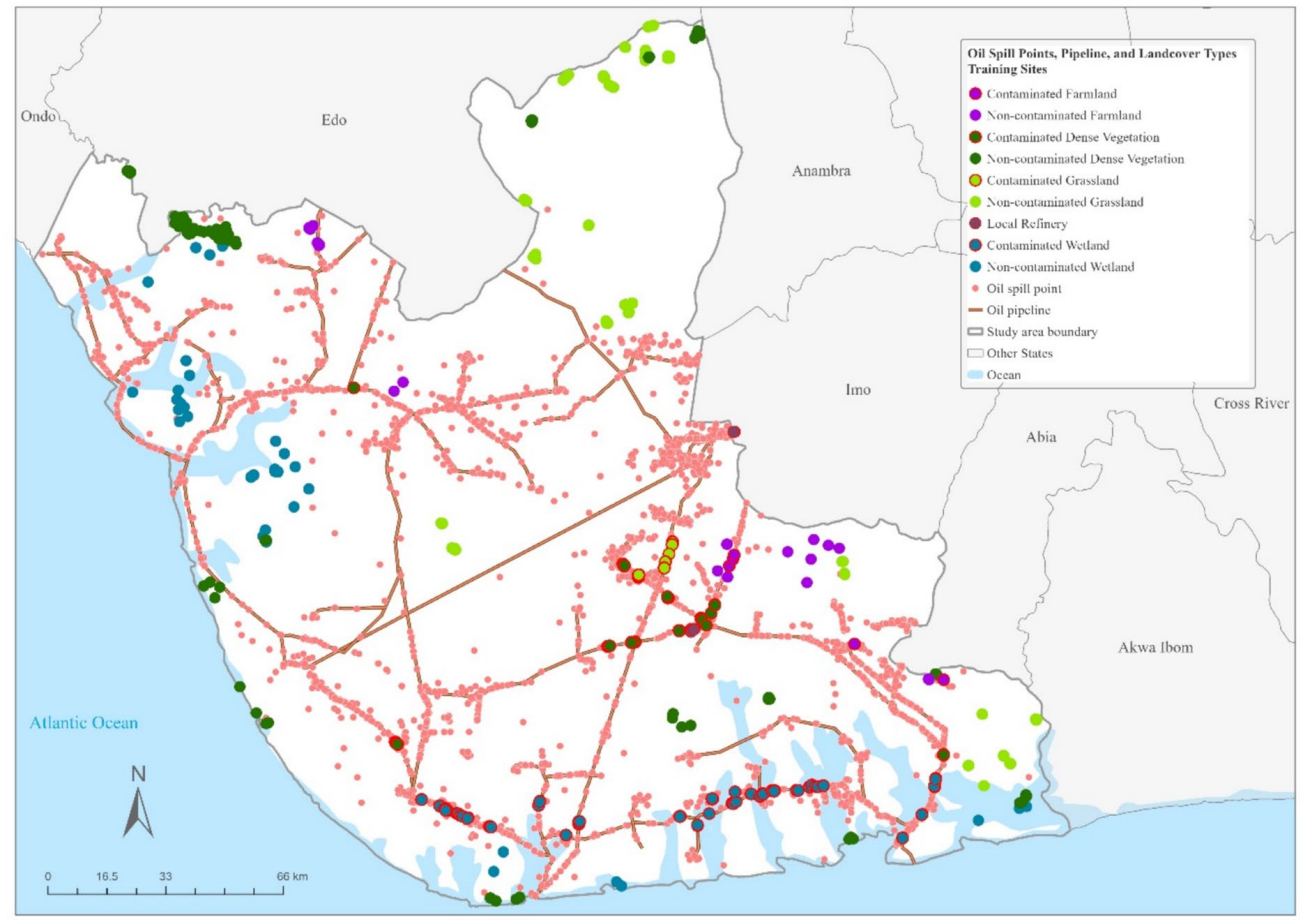
Spatial distribution of oil spill points in relation to oil pipelines and the associated landcover training sites composition (centroids of training sites polygons are displayed for visibility purposes)

Figure 4 illustrates oil spill points in relation to oil pipelines and the composition of associated landcover training sites. This figure is complemented by the summary table (Table 1), which details the extent and representativeness of the selected training sites across different landcover categories. The landcover distribution shows that dense vegetation covers the largest area (22,997.27 km^2^), followed by wetlands (5079.31 km^2^), grasslands (3175.12 km^2^), and farmlands (357.91 km^2^). Training sites were intentionally selected to ensure a balance between contaminated and non-contaminated areas (Table 1), showcasing representation across varying landcover types.

**Table 1:** Representativeness of the selected training sites within each landcover category

Landcover class	Dense vegetation		Wetland		Farmland		Grassland		Local refinery
Total area (km^2^)	22,997.27		5079.31		357.91		3175.12		0.65
Reclassified landcover training site	CDV	NDV	CWL	NWL	CFL	NFL	CGL	NGL	Local refinery
Training area (km^2^)	1.46	123.31	7	66.88	0.05	24.89	0.1	12.96	0.09

*CDV* Contaminated Dense Vegetation, *NDV* Non-Contaminated Dense Vegetation, *CFL* Contaminated Farmland, *NFL* Non-Contaminated Farmland, *CGL* Contaminated Grassland, *NGL* Non-Contaminated Grassland, *CWL* Contaminated Wetland, *NWL* Non-Contaminated Wetland

To assess the discriminative power between contaminated and non-contaminated landcover types, the Jeffries-Matusita (JM) distance was calculated. The JM distance is a statistical measure of dissimilarity or separability between two probability distributions, particularly useful in classification tasks to evaluate class separability. It ranges from 0 (indicating maximum overlap or similarity) to 2 (indicating complete separation). This method is widely employed to assess the effectiveness of feature selection or dimensionality reduction techniques in enhancing class distinction (Aswin, 2024; Zhang et al., 2023).

The JM distance is given by the following:

1 $$JM=2\left(1-exp\left(\frac{-B}{8}\right)\right)$$

where *B* is the Bhattacharyya distance. The Bhattacharyya distance, in turn, is computed as:

2 $$B=\frac{1}{8}{\left({\upmu }_{i}-{\upmu }_{j}\right)}^{T}{\left(\frac{{\Sigma }_{i}+{\Sigma }_{j}}{2}\right)}^{-1}\left({\upmu }_{i}-{\upmu }_{j}\right)+\frac{1}{2}\text{ln}\left(\frac{\left|{\Sigma }_{i}+{\Sigma }_{j}\right|}{2\sqrt{\left|{\Sigma }_{i}\right|\left|{\Sigma }_{j}\right|}}\right)$$

where

$\mu_i$ and $\mu_j$ are the mean reflectance values of two classes $i$ and $j$.

$\textstyle\sum_i$ and $\textstyle\sum_j$ are the covariance matrices (based on standard deviations) for the spectral bands.

For each pair of contaminated and non-contaminated landcover classes, we extracted the following:

(i) Mean reflectance values $\left({\upmu }_{i}-{\upmu }_{j}\right)$ across the bands (blue, green, red, and near infrared) and
(ii) The standard deviations for each band were used to construct diagonal covariance matrices ${(\Sigma }_{i},{\Sigma }_{j})$ of both classes, assuming no correlation between bands.

The JM distance for the landcovers in this study was computed using Python (version 3.7.2).

#### Retrieval of vegetation health indices from PlanetScope data

VHIs using the red, green, blue, and near-infrared (NIR) bands were used to examine the impact of pollutant stressors on vegetation (see Table 2). To generate multiple indices, the study utilised the Awesome Spectral Indices, a standardised, ready-to-use curated list of spectral indices that can be used as expressions for computing spectral indices in GEE (https://github.com/awesome-spectral-indices/awesome-spectral-indices). The training sites were used as area of interests, and the Awesome Spectral Indices parameter was applied to generate the VHIs.

**Table 2:** Extracted vegetation health indices

Vegetation indices	Formula	Source
Green optimised soil adjusted vegetation index (GOSAVI)	(N − G)/(N + G + 0.16)	Sripada et al. (2005)
Green–red normalised difference vegetation index (GRNDVI)	(N − (G + R))/(N + (G + R))	Wang et al. (2022)
Green normalised difference vegetation index (GNDVI)	(N − G)/(N + G)	Gitelson et al. (1996)
Normalised difference vegetation index (NDVI)	(N − R)/(N + R)	Tucker(1979)
Soil-adjusted vegetation index (SAVI)	(1.0 + L) × (N − R)/(N + R + L)	Huete (1988)
Simple ratio (SR)	N/R	Jordan (1969)
Simple ratio (800 and 550 nm) (SR2)	N/G	Buschmann and Nagel (1993)
Two-band enhanced vegetation index (EVI2)	g × (N − R)/(N + 2.4 × R + L)	Jiang et al. (2008)

*R* red, *G* green, *N* near infrared, *B* blue (bands)

*g* gain factor (e.g. used for EVI),

*L* canopy background adjustment (e.g. used for SAVI and EVI)

#### Simple moving average regression

The regression trend analysis approach (Mancino et al., 2022) was adopted to understand the vegetation health in the study area. This approach is particularly useful when dealing with a large number of observations, such as long time series or data collected on a daily or weekly basis. By excluding outliers, the regression trend helps identify the slope of the Line, which is crucial for assessing vegetation health trends. To implement this approach, VHI values generated from 2016 to 2023 were exported from GEE and imported into Python 3.12.6.

#### Machine learning supervised classification

Random forests are groups of decision trees that all use the same random vector to make predictions (Breiman, 2001). As a random forest grows, the error in its generalisation tends to get smaller. Generalisation error is based on how strong the trees are and how well they fit together. Random forests use uncertainty to make classifiers and regression models better. By measuring the strength and correlation of the predictors, the out-of-bag estimate can show how well the random forest can predict (Breiman, 2001). The RF classification was used to distinguish and effectively characterise parts of the landcovers impacted by oil pollution from oil-free parts. The analysis was carried out using the classifiers in Earth Engine API named, ee.Classifier.smileRandomForest(). The smile part refers to the Statistical Machine Intelligence and Learning Engine (SMILE) JAVA library which is used by GEE to implement these algorithms (Gandhi, 2021). The PlanetScope data was accessed and processed through GEE. The input features for the classifier comprised of the spectral bands and extracted vegetation health indices. As recommended by Löw et al. (2021), the inclusion of vegetation indices can significantly improve the accuracy of oil spill detection. Table 3 expands on the classification schema used in this study by defining the various prioritised landcover types. These landcover types were derived from the Sentinel-2 10 m land use/land cover map.

**Table 3:** Classified landcover class definition

Class	Definitions
Water	Areas where water was predominantly present
Contaminated dense vegetation (CDV)	Dense vegetation, typically with a closed or dense canopy where oil spills have been recorded, when the oil spills data was overlayed on the high-resolution image, the NOSDRA oil spill oil spill points were found in this part of the landcover
Non-contaminated dense vegetation (NDV)	Dense vegetation, typically with a closed or dense canopy where there are no oil spills
Contaminated wetland vegetation (CWL)	Areas of vegetation showing evident intermixing with water, despite being contaminated with oil spills, are identified through the presence of recorded oil spill incidents when overlaying the NOSDRA oil spill data onto the landcover
Non-contaminated wetland vegetation (NWL)	Areas of vegetation showing evident intermixing with water, lacking any observed oil spill points within them
Contaminated farmlands/crops (CFL)	Human planted/plotted land area with oil spills point observed within them
Non-contaminated farmlands/crops (NFL)	Human planted/plotted land area with no oil spill points
Contaminated grasslands (CGL)	Open areas covered in homogenous grasses with little to no taller vegetation with oil spill points observed within them
Non-contaminated grasslands (NGL)	Open areas covered in homogenous grasses with little to no taller vegetation with no oil spill points
Local refinery	Site utilised for local, rudimentary crude oil refining

The analysis focused on the image acquired on January 1, 2023, which was deliberately selected to provide sufficient time for the environmental impacts of oil spills to become evident. This date aligns with the temporal scope of oil spill data acquired from the NOSDRA, which extends up to January 2022. By examining the choice of an image from 1 year later ensures that the dataset captures the full extent of the oil spills’ effects on the environment. This 1-year gap provides sufficient time for the ecological consequences, particularly on vegetation and land cover, to manifest, offering a clear basis for classifying impacted areas in the study. The image was clipped to the study area boundary, and to ensure comparability of features for machine learning, all image bands were normalised to a 0–1 range. Multiple feature collections representing different land cover types were merged into a single dataset using the*.merge* function in GEE. Pixel values and corresponding land cover labels were extracted from the image at specified locations within these feature collections. The resulting dataset was then randomly divided into training (70%) and validation (30%) sets for model development and assessment. A random forest classifier with 300 trees was trained using the prepared training data.

To evaluate the accuracy of the classification, an accuracy assessment was performed using the *ee.Classifier.confusionMatrix()* method in GEE. A fivefold cross-validation was performed in GEE by partitioning the data into five stratified folds in a random manner using the utils package, with the objective of preserving a consistent ratio of all classes in each fold. In each iteration of the classification, a single data fold is reserved for testing purposes while the remaining k-1 folds are utilised for training the classifier. The classifier’s performance quality was evaluated on the excluded fold in each experiment, and subsequently, the overall performance metric was averaged across the k-folds. The results of the classification were exported to ArcGIS Pro 3.3 to calculate the spatial extent of the different classes and for visualisation.

## Results and discussion

### Analysis of contaminated landcover against non-contaminated landcover

To understand the separability of the contaminated against non-contaminated landcovers, the computed Jeffries-Matusita (JM) distances were calculated (see Table 4). For CDV vs NDV, CGL vs NGL, and CWL vs NWL, the JM distances are close to 2, indicating that these pairs are highly separable, meaning contaminated and non-contaminated versions of these landcover types are easily distinguishable based on their spectral profiles. CFL vs NFL has a much lower JM distance (0.17), indicating poor separability. The contaminated and non-contaminated farmlands have very similar spectral profiles, making it difficult to distinguish between them using the four bands.

**Table 4:** Computed Jeffries-Matusita (JM) distances for spectral bands and landcover types

Landcover pair	Computed Jeffries-Matusita (JM) distances
CDV vs NDV	1.99
CFL vs NFL	0.17
CGL vs NGL	1.99
CWL vs NWL	1.97

**Fig. 5:**
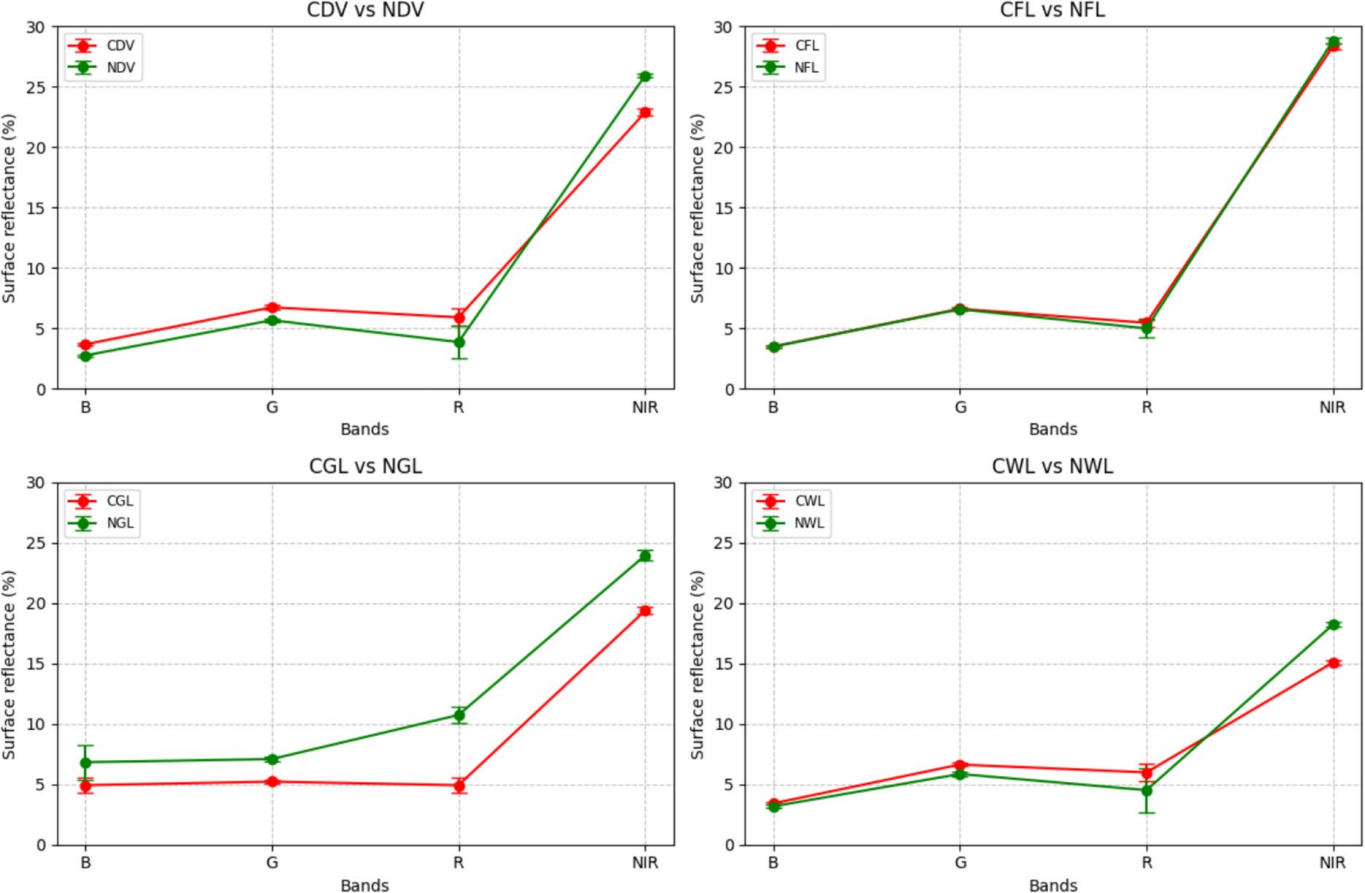
Cross-analysis of the contaminated landcover against non-contaminated landcover

In comparing CDV to NDV, the spectral reflectance values across various bands showcase discernible differences. Apart from the NIR band, CDV consistently displays higher reflectance. Specifically, in the blue band, CDV demonstrates a reflectance of 3.7% with a standard deviation of ± 2.0%, contrasting with NDV’s 2.7% (± 0.5%). This pattern continues in the green band (CDV, 5.8% ± 1.0%; NDV, 5.7% ± 0.5%) and the red band (CDV, 5.5% ± 1.0%; NDV, 3.8% ± 0.5%). However, in the NIR band, the trend reverses, with NDV showing a higher reflectance (25.5% ± 1.0%) than CDV (23.5% ± 2.0%) (see Fig. 5).

Moving to CFL versus NFL, the reflectance values show much greater similarity across the spectrum. In the blue band, their values are nearly identical, with CFL at 3.6% (± 0.5%) and NFL at 3.5% (± 0.4%). This close correspondence persists in the green band (CFL, 6.8% ± 0.8%; NFL, 6.7% ± 0.6%) and the red band (CFL, 5.3% ± 1.0%; NFL, 4.9% ± 0.6%). Even in the NIR band, the separation remains minimal (CFL, 28.5% ± 1.0%; NFL, 28.2% ± 1.0%), indicating that these two land cover types are spectrally very similar.

Contrasting CGL with NGL reveals a clear and significant distinction in spectral reflectance, with NGL consistently exhibiting higher values across all bands. For instance, in the blue band, NGL shows a reflectance of 6.8% (± 1.9%), whereas CGL displays a lower 4.9% (± 1.9%). This trend is even more pronounced in the red band (NGL, 10.8% ± 1.0%; CGL, 4.9% ± 1.0%) and the NIR band (NGL, 24.0% ± 1.0%; CGL, 19.5% ± 2.0%), indicating a strong and consistent spectral separation between the two land cover types.

Lastly, when comparing CWL to NWL, CWL generally displays higher reflectance values in the visible spectrum, but this pattern reverses in the NIR region. In the blue band, CWL exhibits a reflectance of 3.4% (± 1.3%), slightly higher than NWL’s 3.2% (± 1.2%). This pattern continues in the green band (CWL, 6.5% ± 1.0%; NWL, 6.2% ± 1.0%) and red band (CWL, 6.0% ± 1.0%; NWL, 4.8% ± 2.0%). In the NIR band, however, NWL shows a notably higher reflectance (18.0% ± 1.5%) compared to CWL (15.0% ± 2.0%). This crossover in the NIR band is a key distinguishing feature between these two land cover types.

### Spectral separability of the vegetation health indices

**Fig. 6:**
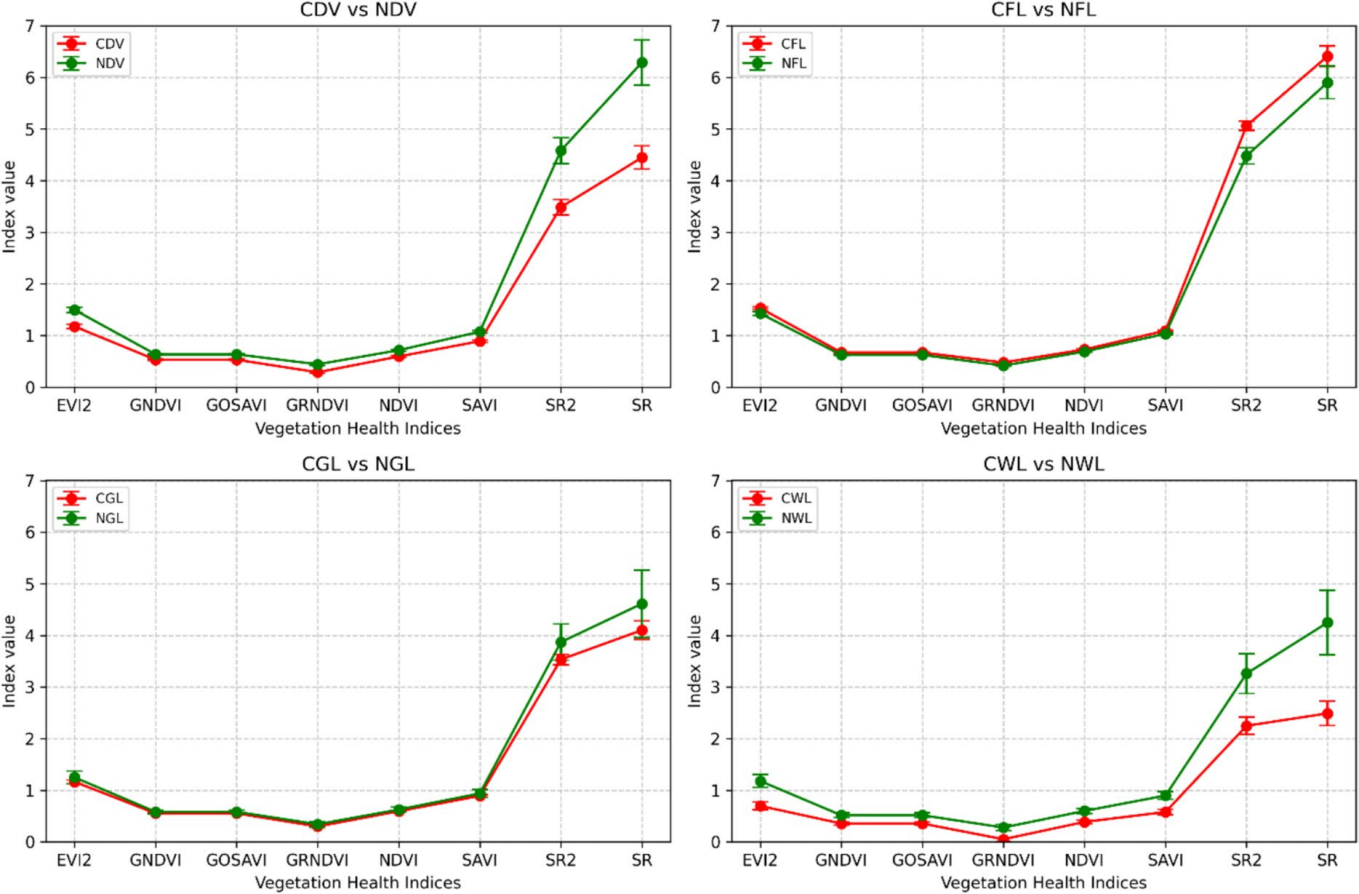
Spectral separability between different landcover types, primarily based on their vegetation health indices

To enhance the classification of contaminated landcovers from the non-contaminated landcovers, various vegetation indices were incorporated into the classifier. Figure 6 facilitates the visual comparison of vegetation health indices between the landcover types. The analysis reveals varying degrees of spectral separability between the landcover types, primarily based on their vegetation health indices. The calculated JM distances indicate varying degrees of separability between contaminated and non-contaminated land covers across different vegetation indices. The simple ratio indices (SR and SR2) emerged as the most powerful discriminators (see Table 5). For example, SR shows a strong separation of about 1.46 when separating CDV from NDV and maintains a robust 1.23 value for CWL landcover versus its NWL counterpart. SR2 performs closely, with values exceeding 1.0 for the most easily separated pairs and around 0.80 for those that are slightly distinguish. Following these, EVI2 stands out as the next most robust index, yielding a moderate separation (around 0.19) even in the difficult-to-distinguish cases, such as CGL versus NGL, where other indices show minimal distinction (barely past 0.15). At the lower end of the spectrum, GNDVI, GOSAVI, and GRNDVI offer only marginal discrimination, with JM distances typically between 0.12 and 0.27 across all pairs. NDVI and SAVI fall into a middle category; while useful, their separability does not exceed 0.40. The high JM distances in certain pairs underscore the potential utility of these vegetation indices for classifying landcover contamination, while the lower distances suggest that additional features or indices may be needed to improve separability in certain cases.

**Table 5:** Computed Jeffries-Matusita (JM) distances for the VHIs and landcovers

Index	CDV vs NDV	CFL vs NFL	CGL vs NGL	CWL vs NWL
SR	1.46	0.26	0.33	1.23
SR2	1.07	0.60	0.29	0.80
EVI2	0.48	0.11	0.19	0.53
SAVI	0.30	0.08	0.18	0.38
GRNDVI	0.25	0.10	0.17	0.27
NDVI	0.20	0.06	0.18	0.26
GNDVI	0.16	0.12	0.15	0.18
GOSAVI	0.16	0.12	0.15	0.18

### Trend analysis

To assess the health trend of vegetation in areas affected by oil spills, the dense vegetation, wetland, grassland, and farmland (both contaminated and non-contaminated) were analysed using SR, SR2, EVI2, NDVI, GRNDVI, GNDVI, and GOSAVI Spearman’s rank correlation (ρ) between each vegetation-health index and year (2016–2023) for contaminated vs. non-contaminated landcovers was computed, and the statistical significance (*P* < 0.05) was assessed.

**Fig. 7:**
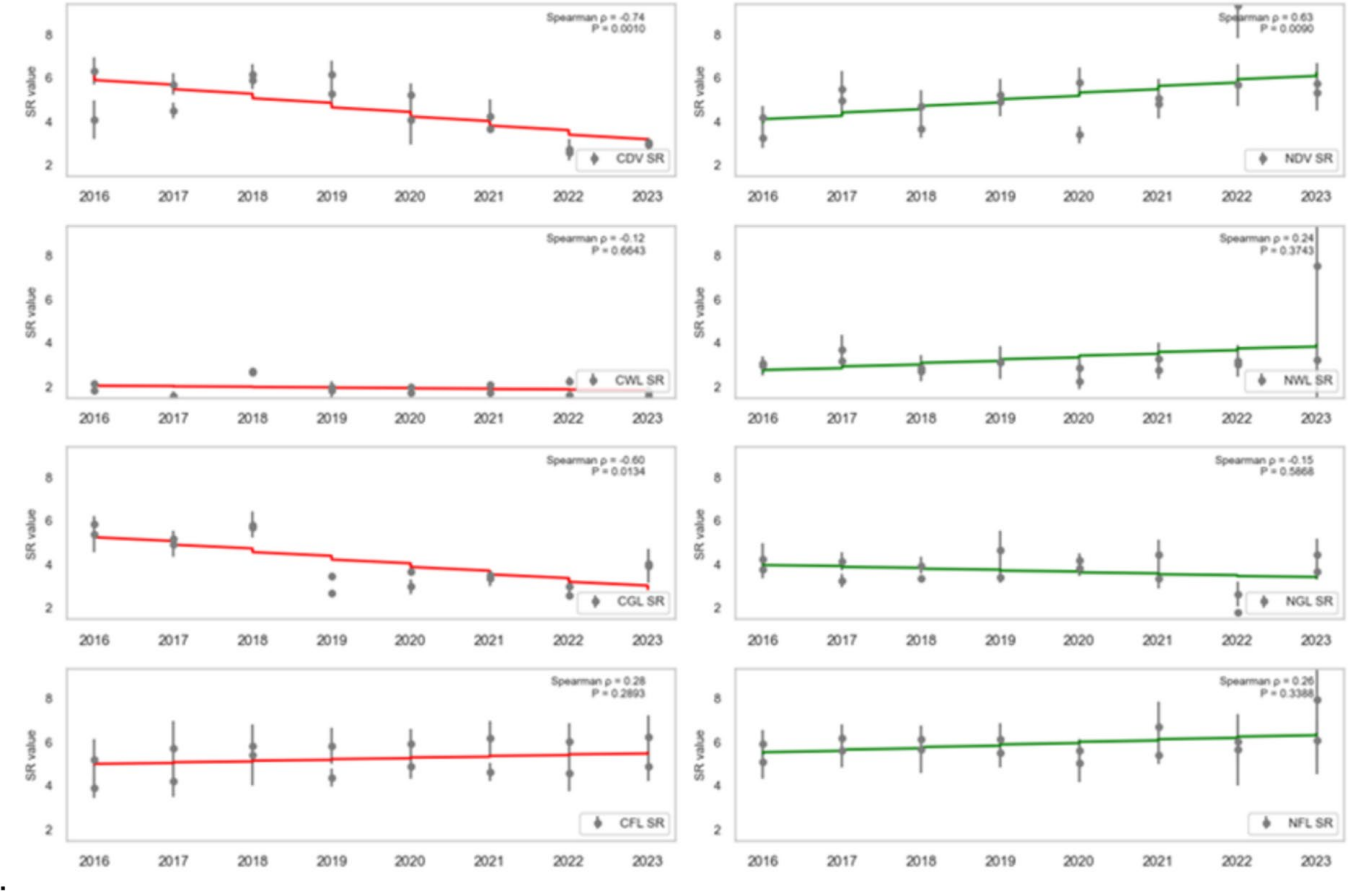
SR trends of contaminated and non-contaminated landcover types from 2016 to 2023 (the red lines represent the trends of contaminated landcovers while the green lines represent the non-contaminated counterparts)

**Fig. 8:**
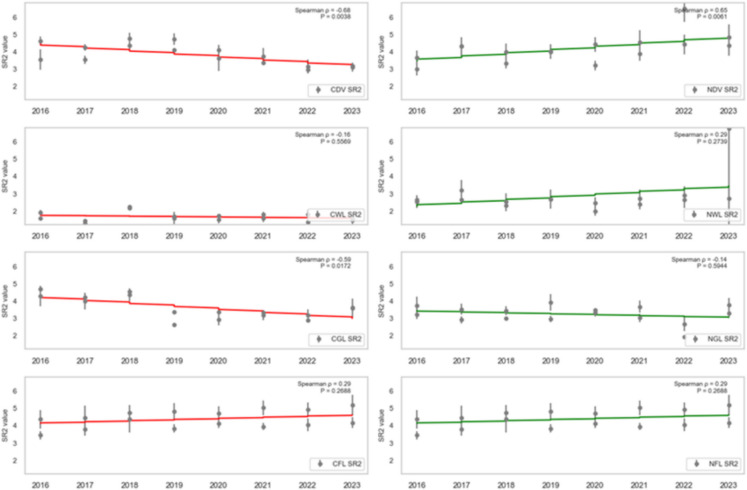
SR2 trends of contaminated and non-contaminated landcover types from 2016 to 2023 (the red lines represent the trends of contaminated landcovers while the green lines represent the non-contaminated counterparts)

**Fig. 9:**
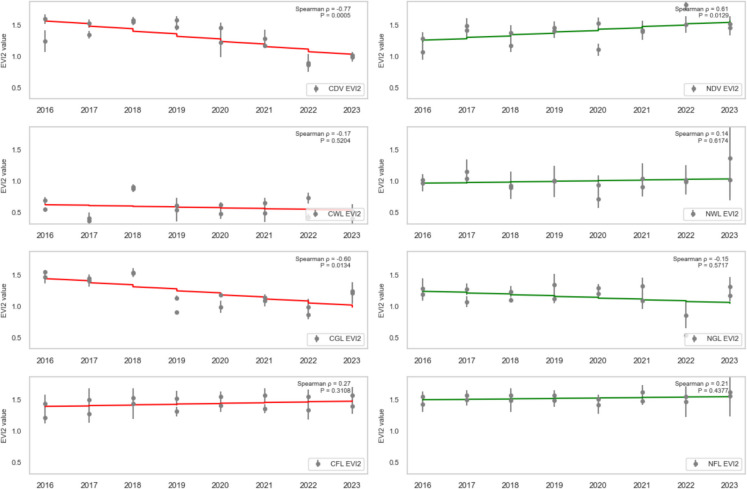
EVI2 trends of contaminated and non-contaminated landcover types from 2016 to 2023

**Fig. 10:**
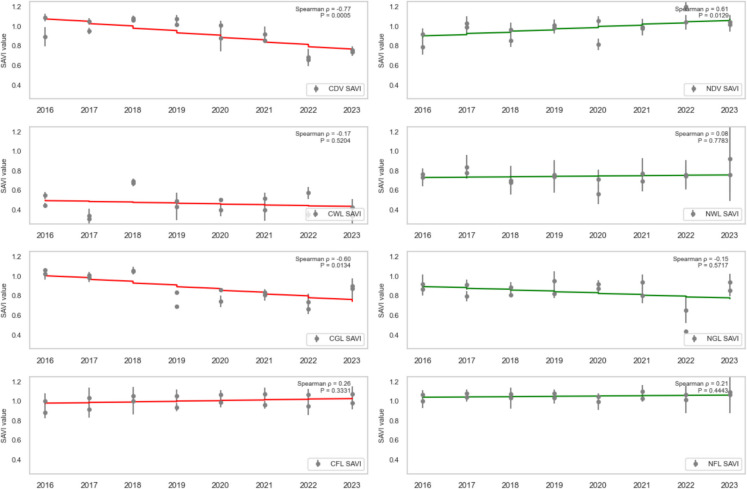
SAVI trends of contaminated and non-contaminated landcover types from 2016 to 2023

**Fig. 11:**
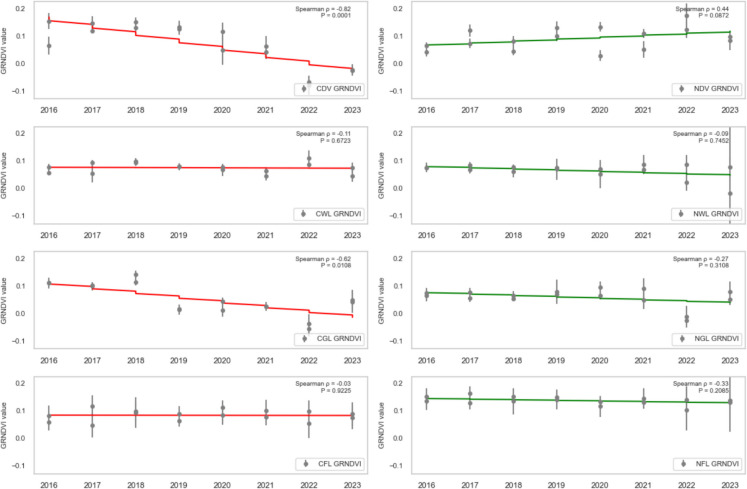
GRNDVI trends of contaminated and non-contaminated landcover types from 2016 to 2023

**Fig. 12:**
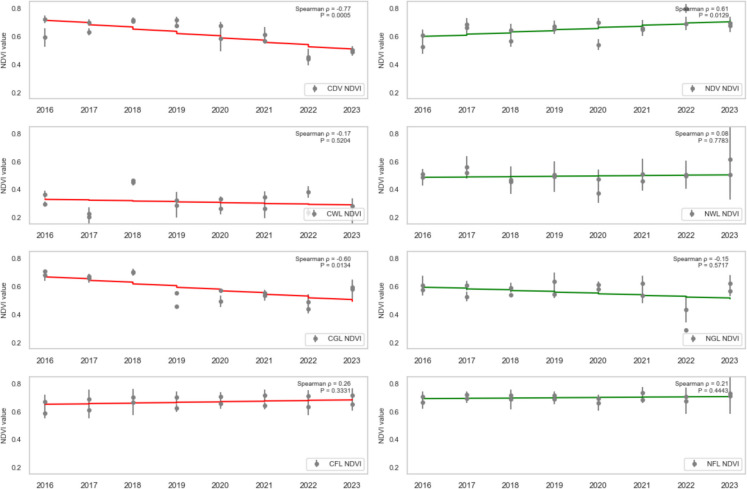
NDVI trends of contaminated and non-contaminated landcover types from 2016 to 2023

**Fig. 13:**
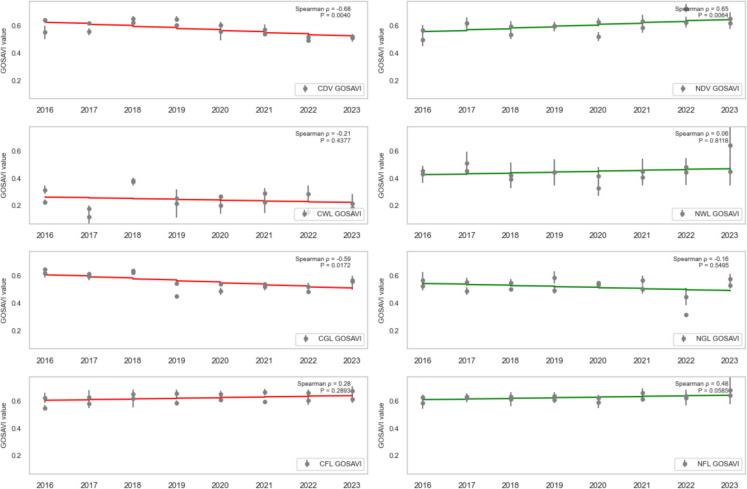
GOSAVI trends of contaminated and non-contaminated landcover types from 2016 to 2023

**Fig. 14:**
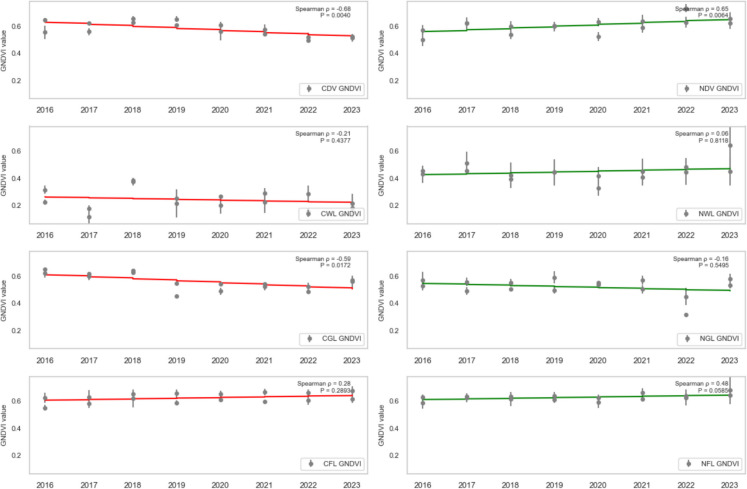
GNDVI trends of contaminated and non-contaminated landcover types from 2016 to 2023

CDV landcover exhibited a strong, statistically significant decline across every vegetation index from 2016 to 2023, reflecting severe degradation due to oil spill impacts. Specifically, CDV SR (standardised slope = − 0.74, *P* = 0.0010; Fig. 7), SR2 (standardised slope = − 0.68, *P* = 0.0038; Fig. 8), EVI2 (standardised slope = − 0.77, *P* = 0.0005; Fig. 9), SAVI (standardised slope = − 0.77, *P* = 0.0005; Fig. 10), GRNDVI (standardised slope = − 0.82, *P* = 0.0001; Fig. 11), NDVI (standardised slope = − 0.77, *P* = 0.0005; Fig. 12), GNDVI (standardised slope = − 0.68, *P* = 0.0040; Fig. 13), and GOSAVI (standardised slope = − 0.68, *P* = 0.0040; Fig. 14) all show steep, significant downward trends. These consistent negative trends confirm a pronounced, multi-index deterioration of vegetation health in CDV.

By contrast, NDV landcover demonstrates significant positive trends in every index, indicating improving or stable vegetation in uncontaminated areas. NDV SR (standardised slope = + 0.63, *P* = 0.0090; Fig. 7), SR2 (standardised slope = + 0.65, *P* = 0.0061; Fig. 8), EVI2 (standardised slope = + 0.61, *P* = 0.0129; Fig. 9), SAVI (standardised slope = + 0.61, *P* = 0.0129; Fig. 10), GRNDVI (standardised slope = + 0.44, *P* = 0.0872; Fig. 11), NDVI (standardised slope = + 0.61, *P* = 0.0129; Fig. 12), GNDVI (standardised slope = + 0.65, *P* = 0.0064; Fig. 13), and GOSAVI (standardised slope = + 0.65, *P* = 0.0064; Fig. 14) all trend upward. These metrics highlight healthy and improving vegetation conditions in NDV, likely due to favourable environmental conditions or the absence of oil contamination, serving as a positive reference.

In CWL areas, no index shows a statistically significant trend, though all eight indices exhibit slight downward slopes. CWL SR (standardised slope = − 0.12, *P* = 0.6643; Fig. 7), SR2 (standardised slope = − 0.16, *P* = 0.5569; Fig. 8), EVI2 (standardised slope = − 0.17, *P* = 0.5204; Fig. 9), SAVI (standardised slope = − 0.17, *P* = 0.5204; Fig. 10), GRNDVI (standardised slope = − 0.11, *P* = 0.6723; Fig. 11), NDVI (standardised slope = − 0.17, *P* = 0.5204; Fig. 12), GNDVI (standardised slope = − 0.21, *P* = 0.4377; Fig. 13), and GOSAVI (standardised slope = − 0.21, *P* = 0.4377; Fig. 14) suggest a gentle decline but remain statistically indistinguishable from. This suggests potential resilience of wetland vegetation to oil spills, possibly due to the waterlogged environment.

NWL landcover remains remarkably stable, with all indices showing weak, non-significant trends around zero. NWL SR (standardised slope = + 0.24, P = 0.3743; Fig. 7), SR2 (standardised slope = + 0.29, *P* = 0.2739; Fig. 8), EVI2 (standardised slope = + 0.14, *P* = 0.6174; Fig. 9), SAVI (standardised slope = + 0.08, *P* = 0.7783; Fig. 10), GRNDVI (standardised slope = − 0.09, *P* = 0.7452; Fig. 11), NDVI (standardised slope = + 0.08, *P* = 0.7783; Fig. 12), GNDVI (standardised slope = + 0.06, *P* = 0.8118; Fig. 13), and GOSAVI (standardised slope = + 0.06, *P* = 0.8118; Fig. 14) indicate that the NWL region remains relatively stable and serves as a baseline for comparison.

CGL landcover exhibited strong downward trends across all indices, mirroring CDV but at moderate strength. CGL SR (standardised slope = − 0.60, *P* = 0.0134; Fig. 7), SR2 (standardised slope = − 0.59, *P* = 0.0172; Fig. 8), EVI2 (standardised slope = − 0.60, *P* = 0.0134; Fig. 9), SAVI (standardised slope = − 0.60, *P* = 0.0134; Fig. 10), GRNDVI (standardised slope = − 0.62, *P* = 0.0108; Fig. 11), NDVI (standardised slope = − 0.60, *P* = 0.0134; Fig. 12), GNDVI (standardised slope = − 0.59, *P* = 0.0172; Fig. 13), and GOSAVI (standardised slope = − 0.59, *P* = 0.0172; Fig. 14) all show clear downward trends. NGL shows no significant trends, indicating stable but slow recovery.

In contrast, NGL landcover shows no significant trends in any index. NGL SR (standardised slope = − 0.15, *P* = 0.5868; Fig. 7), SR2 (standardised slope = − 0.14, *P* = 0.5944; Fig. 8), EVI2 (standardised slope = − 0.15, *P* = 0.5717; Fig. 9), SAVI (standardised slope = − 0.15, *P* = 0.5717; Fig. 10), GRNDVI (standardised slope = − 0.27, *P* = 0.3108; Fig. 11), NDVI (standardised slope = − 0.15, *P* = 0.5868; Fig. 12), GNDVI (standardised slope = − 0.16, *P* = 0.5495; Fig. 13), and GOSAVI (standardised slope = − 0.16, *P* = 0.5495; Fig. 14) suggest a lack of measurable change.

Despite oil-spill presence, CFL indices remain flat to mildly positive, none reaching statistical significance. CFL SR (standardised slope = + 0.28, *P* = 0.2893; Fig. 7), SR2 (standardised slope = + 0.29, *P* = 0.2688; Fig. 8), EVI2 (standardised slope = + 0.27, *P* = 0.3108; Fig. 9), SAVI (standardised slope = + 0.26, *P* = 0.3331; Fig. 10), GRNDVI (standardised slope = + 0.03, *P* = 0.9225; Fig. 11), NDVI (standardised slope = + 0.26, *P* = 0.3331; Fig. 12), GNDVI (standardised slope = + 0.28, *P* = 0.2893; Fig. 13), and GOSAVI (standardised slope = + 0.28, *P* = 0.2893; Fig. 14) suggest limited degradation, possibly buffered by agricultural activities. NFL also shows modest upward tendencies across all indices without significant declines. NFL SR (standardised slope = + 0.26, *P* = 0.3388; Fig. 7), SR2 (standardised slope = + 0.29, *P* = 0.2688; Fig. 8), EVI2 (standardised slope = + 0.21, *P* = 0.4432; Fig. 9), SAVI (standardised slope = + 0.21, *P* = 0.4432; Fig. 10), GRNDVI (standardised slope = − 0.33, *P* = 0.2085; Fig. 11), NDVI (standardised slope = + 0.21, *P* = 0.4443; Fig. 12), GNDVI (standardised slope = + 0.48, *P* = 0.0585; Fig. 13), and GOSAVI (standardised slope = + 0.48, *P* = 0.0583; Fig. 14) indicate a trend toward vegetation improvement.

This multi-index analysis underscores the pronounced impact of oil spills on contaminated regions such as CDV and CGL, and highlights stability or improvement in non-contaminated areas (NDV, NWL, NFL), reinforcing the value of using multiple vegetation indices to assess environmental health.

### Accuracy assessment

A confusion matrix was used to evaluate the performance of the classification model by comparing predicted and actual class labels. The test accuracy value, 0.98, indicates the overall accuracy of the classification model on the test dataset. It represents the proportion of correctly predicted instances out of the total number of instances in the test dataset.

To further verify the model, a k-fold cross-validation was conducted. Results from the cross-validation (k-fold) show that the training errors range from 0.9996 to 0.9995 in all the folds, indicating a high accuracy of the model on the training data (see Table 6). The validation errors (OOB) range from 0.9894 to 0.9877, suggesting a slightly lower accuracy on the validation data compared to the training data. The mean error is 0.9886, which is the average validation error across the different iterations.

**Table 6:** K-fold cross validation results

Fold	Training error	Validation error
1	0.9996	0.9893
2	0.9995	0.9883
3	0.9997	0.9877
4	0.9996	0.9894
5	0.9995	0.9882

### Spatial distribution of contaminated sites

**Fig. 15:**
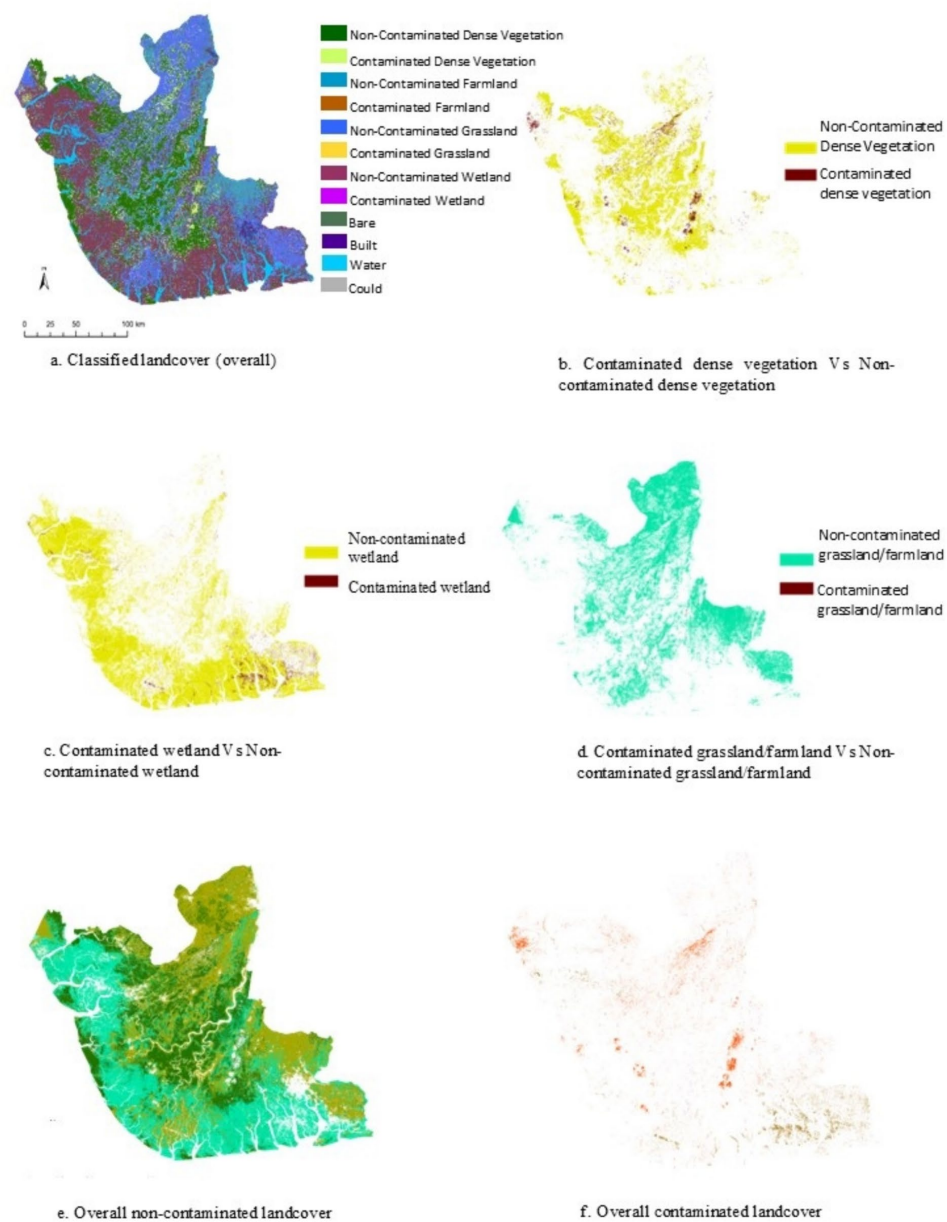
Thematic classification outputs displaying the spatial extent and corresponding area in hectares of different landcovers generated from January 2023 PlanetScope data

The classification results presented in Fig. 15 provide information on the spatial extent of different landcovers generated from the January 2023 PlanetScope data, while Table 7 provides the corresponding area of the different landcovers in hectares. Figure 15e and f specifically display the spatial extent of non-contaminated dense vegetation and contaminated vegetation. Table 7 shows that the non-contaminated dense vegetation covers 22.8% of the total landcover area, which is equivalent to 8390 ha. On the other hand, the contaminated dense vegetation, affected by oil spills, constitutes 1.39% of the total landcover area, amounting to 513 ha across the three states (Bayelsa, Delta, and Rivers). The non-contaminated wetland accounts for 35.4% of the total landcover area, encompassing 13,023 ha. Conversely, the wetland area contaminated by oil spills represents 1.7% of the total landcover area, totaling 625 ha. To enhance visual clarity on the map, the grassland and farmland classes were merged.

**Table 7:** Landcover area estimates

Landcover	Area (hectares)	Area (%)
Non-contaminated dense vegetation	8390	22.81%
Contaminated dense vegetation	513	1.39%
Non-contaminated farmland	3326	9.04%
Contaminated farmland	26	0.07%
Non contaminated grassland	7475	20.32%
Contaminated grassland	13	0.04%
Local refinery	2	0.01%
Non-contaminated wetland	13,023	35.40%
Contaminated wetland	625	1.70%
Bare	365	0.99%
Built	532	1.45%
Water	2471	6.72%
Clouds	24	0.07%

The results reveal that the non-contaminated grass/farmland covers 29.4% of the total landcover area, equivalent to 10,801 ha. Meanwhile, the grassland/farmland contaminated by oil spills occupies a minimal portion of the landcover, constituting only 0.11% or 39 ha. Overall, the prioritised landcovers, including contaminated vegetation, wetland, farmland, grassland, and local refinery locations, cover approximately 4% (1180 ha) of the total area. Conversely, the non-contaminated prioritised landcovers (non-contaminated vegetation, wetland, farmland, and grassland) account for 96% (32,215 ha) of the total landcover area.

### Discussion

The Niger Delta, a globally significant biodiversity hotspot, faces severe environmental degradation due to recurrent oil spills, significantly impacting vegetation health and ecosystem integrity (NDDC, n.d.; Obi, 2023). Vegetation indices derived from remote sensing data have proven effective in differentiating hydrocarbon-contaminated from non-contaminated soils (Ansah et al., 2022; Huete, 2004). However, traditional remote sensing approaches have struggled with accuracy in the humid tropics due to cloud cover, data availability, and algorithmic limitations (Adamu et al., 2018). To address these challenges, this study developed a remote sensing approach focused on spectral separability, temporal vegetation health trends, and spatial mapping of oil-contaminated areas.

Spectral analysis demonstrated clear separability between contaminated and non-contaminated vegetation, notably in dense vegetation, grassland, and wetlands, indicated by high Jeffries-Matusita (JM) distances. Farmland, however, exhibited poor separability, possibly due to soil background reflectance and varying agricultural practices (Al-Shammary et al., 2024; Prudnikova et al., 2019). Although healthy vegetation typically shows lower red band reflectance compared to stressed or contaminated vegetation (Adamu et al., 2015; Lassalle et al., 2020), our results indicated higher red reflectance in non-contaminated grassland. This may be due to additional stress at the control site or the influence of darker, oil-affected soil background masking the reflectance of contaminated vegetation (Lassalle, 2020). VHIs such as SR, SR2, EVI2, GNDVI, SAVI, GRNDVI, and NDVI further enhanced the separability, supporting their effectiveness for identifying oil-induced vegetation stress (Adamu et al., 2018; Ansah et al., 2022). Among these, the separability analysis highlighted SR and SR2 as the most effective indices for distinguishing oil-contaminated vegetation from non-contaminated classes.

Temporal analysis (2016–2023) revealed significant declines in VHIs for contaminated dense vegetation and grassland, indicating persistent vegetation degradation due to chronic oil pollution. Wetlands showed only moderate declines, suggesting greater resilience potentially due to hydrological and biological buffering effects (Breil et al., 2022; Dordio et al., 2008). Farmland exhibited an unexpected flat-to-upward VHI trend, possibly influenced by agricultural practices that mitigate surface oil concentrations, although this finding likely masks severe localised degradation (Ratcliffe, 2019; Chinedu & Chukwuemeka, 2018).

Spatial mapping confirmed that contamination disproportionately affects dense vegetation and wetlands, with contamination significantly concentrated along waterways. In contrast, farmland and grassland showed localised contamination Linked directly to infrastructure proximity. Overall, contaminated areas represented approximately 4% of the landscape, underscoring the need for targeted monitoring and remediation strategies. These findings provide insights for managing environmental impacts in the Niger Delta, highlighting the strengths and limitations of remote sensing for monitoring oil spill–induced ecological degradation.

This study, while providing valuable insights into the impacts of oil spills, acknowledges several limitations stemming from the characteristics of the satellite data and the methodological approach. The use of PlanetScope NICFI data, with only four spectral bands, restricted the ability to detect subtle changes and specific contamination signatures, especially in diverse land covers. The reliance on bi-annual and monthly composites introduced temporal smoothing, limiting detection of rapid or seasonal ecological changes. Additionally, using only the Random Forest algorithm may have constrained the analysis; future studies could benefit from comparing multiple classification methods. The lack of ground-truth data also limits the validation of remote sensing results, though this is often unavoidable in challenging environments like the Niger Delta. Addressing these limitations with higher spectral and temporal resolution data, varied analytical approaches, and field validation would strengthen future research on oil spill impacts.

## Conclusions

The efficacy of integrating cloud computing and machine learning classification algorithms for vegetation health trend analysis and to assess change dynamics across Bayelsa, Delta and River states in Niger Delta Region has been demonstrated in this study. Analysis of Vegetation Health Indices (VHIs) derived from PlanetScope data (2016–2023) revealed significant impacts of oil spills on vegetation health across different land cover types. The most pronounced decline in VHIs occurred in contaminated dense vegetation, indicating severe stress and degradation. Contaminated grassland also showed a significant downward trend, highlighting its vulnerability, while contaminated wetlands exhibited a more complex response with a slight VHI decrease, suggesting a less severe impact compared to the other contaminated types.

Spatial analysis quantified land cover, showing non-contaminated areas dominate (96%), while contaminated landcovers (wetlands, dense vegetation, farmland/grassland, and local refineries) constitute approximately 4%. The land cover estimates obtained have enabled a nuanced understanding of both contaminated and uncontaminated landcovers and reveal the extent of oil pollution within the studied landcovers. The study showcases the utility of these assessment and quantification techniques for disseminating information to local authorities and guiding response efforts, particularly for the heavily impacted landcovers like the dense vegetation and wetlands. By identifying regions requiring immediate attention for mitigating the impact of oil spills on vegetation, these techniques hold promise for enhancing environmental monitoring and compliance in oil-producing regions like Nigeria. The findings underscore the potential of spectral techniques for assessing and monitoring oil spills, thereby aiding in the identification and remediation of polluted sites. There is an urgent need for remote sensing–based approaches in assessing and managing contaminated areas in the Niger Delta. Through the integration of satellite imagery and advanced analytical tools, this study offers valuable insights into the environmental impacts of oil spills, thereby informing decision-making processes aimed at conserving and restoring the delicate ecosystems of the Niger Delta.

## Data Availability

No datasets were generated or analysed during the current study.
